# Annual acknowledgement of manuscript reviewers

**DOI:** 10.1186/1472-6939-14-14

**Published:** 2013-04-23

**Authors:** Adrian Aldcroft

**Affiliations:** 1 BioMed Central, Floor 6, 236 Gray's Inn Road, London WC1X 8HB, UK

## Abstract

**Contributing reviewers:**

The editors of *BMC Medical Ethics* would like to thank all our reviewers who have contributed to the journal in Volume 13 (2012).

## 

Aslihan Akpinar

Turkey

Nurten Aksoy

Turkey

Alessandra Alciati

Italy

Judy Allen

Australia

John Appiah-Poku

Ghana

Kirsten Austad

USA

Jacquineau Azetsop

Chad

Geoff Ball

Canada

Angela Ballantyne

New Zealand

Sandeep Bavdekar

India

Mauricio Besio

Chile

Pierre-Henri Bréchat

France

Annelien L. Bredenoord

Netherlands

David Buchanan

USA

Philippe Calain

Switzerland

Emily Carrier

USA

Santosh Chaturvedi

India

Murat Civaner

Turkey

Lorraine Corfield

United Kingdom

Leonardo De Castro

Singapore

Paola De Castro

Italy

José Geraldo De Freitas Drumond

Brazil

Martine De Vries

Netherlands

Michael Dunn

United Kingdom

Mette Ebbesen

Denmark

Andrew Edgar

United Kingdom

Sarah Edwards

United Kingdom

Carolyn Ells

Canada

Hamza Eskandarani

Saudi Arabia

Zaynab Essack

South Africa

Nir Eyal

USA

Conrad Fernandez

Canada

Maiko Fujimori

Japan

Freda Ganz

Israel

Raul Garza

Mexico

Hugo Geerts

USA

Jessica Gipson

USA

Kw Goodman

USA

Christine Grady

USA

Glenn Griener

Canada

Just Haffeld

Norway

Joerg Halter

Switzerland

Muhammad Hammami

Saudi Arabia

Alice Hawkins Virani

Canada

Shady Hayek

Lebanon

Lorens Helmchen

USA

Oliver Herber

United Kingdom

Nina Horwitz

Chile

J L Hutton

United Kingdom

Joyce Ikingura

Tanzania

Ana Iltis

USA

Rita-Marie Jansen

South Africa

Gernot Kaiser

Germany

Jerome Kassirer

USA

Vedran Katavic

Croatia

Kenneth Katz

USA

Maureen Kelley

USA

Aaron Kesselheim

USA

Sy Kim

USA

Jonathan Kimmelman

Canada

Suzanne Kosmider

Australia

Stephen Latham

USA

James Lavery

Canada

Richard Lessells

United Kingdom

Wendy Lipworth

Australia

Sergio Litewka

USA

Rurik Löfmark

Sweden

Fernando Lolas

Cocos (Keeling) Islands

Daniel López

Spain

John Luque

USA

Phillipa Malpas

New Zealand

Georg Marckmann

Germany

Deborah Mascalzoni

Italy

Zubin Master

USA

Michael Mcdonald

Canada

Heidi Mertes

Belgium

Dominik Michalski

Germany

Cecilia Milford

South Africa

Maurice B Mittelmark

Norway

Catherine Molyneux

Kenya

Elaine Morgan

USA

Erick Nyambedha

Kenya

Kieran O'Doherty

Canada

Kanu Okike

USA

Michael Paasche-Orlow

USA

Malcolm Parker

Australia

Renzo Pegoraro

Italy

Anna-Maija Pietilä

Finland

Raymond Pitetti

USA

Daryl Pullman

Canada

Carmen Radecki Breitkopf

USA

Michael Ramharter

Gabon

Raffaella Ravinetto

Belgium

Kevin Reid

USA

Eduardo Rodriguez

Chile

Susannah Rose

USA

Daniel Rossignol

USA

Laura Rueda Castro

Chile

Raphael Saginur

Canada

Helen Sammons

United Kingdom

John Santelli

USA

Seema Shah

USA

Beth Shaz

USA

Roberta Siliquini

Italy

Henry Silverman

USA

Mohammad Reza Sohrabi

Iran

Iva Sorta-Bilajac

Croatia

Antonio G. Spagnolo

Italy

Joseph Stanford

USA

Gordon Stirrat

United Kingdom

Avinash Supe

India

Paulina Taboada

Chile

Alan Tait

USA

Godfrey Tangwa

Cameroon

Noritoshi Tanida

Japan

Paul Tiffin

United Kingdom

Mark Tomlinson

South Africa

Irene Tuffrey-Wijne

Uruguay

Antonios Tzamaloukas

USA

Lars Ursin

Norway

Daniel Vallero

USA

Steven Van De Walle

Netherlands

Geetika Verma

Canada

Claudio Verusio

Italy

Edgar Whitley

United Kingdom

Guy Widdershoven

Netherlands

Rochelle Yanofsky

Canada

Farzaneh Zahedi

Iran

Daniella Zipkin

USA

Gilbert Zulian

Switzerland

Matjaz Zwitter

Slovenia

## Pre-publication history

The pre-publication history for this paper can be accessed here:

http://www.biomedcentral.com/1472-6939/14/14/prepub

